# Interactive Gene Expression Between *Metarhizium anisopliae* JEF-290 and Longhorned Tick *Haemaphysalis longicornis* at Early Stage of Infection

**DOI:** 10.3389/fphys.2021.643389

**Published:** 2021-05-19

**Authors:** Mi Rong Lee, Jong Cheol Kim, So Eun Park, Se Jin Lee, Woo Jin Kim, Doo-Hyung Lee, Jae Su Kim

**Affiliations:** ^1^Department of Agricultural Biology, College of Agriculture and Life Sciences, Jeonbuk National University, Jeonju, South Korea; ^2^Department of Life Sciences, College of Bionano, Gachon University, Seongnam, South Korea; ^3^Department of Agricultural Convergence Technology, Jeonbuk National University, Jeonju, South Korea

**Keywords:** *Metarhizium anisopliae*, *Haemaphysalis longicornis*, transcription, catabolic process, peptides synthesis

## Abstract

The longhorned tick, *Haemaphysalis longicornis* (Acari: Ixodidae), is a hard tick and a vector for severe fever with thrombocytopenia syndrome (SFTS) virus. The number of patients infected with SFTS is rapidly increasing. Recently, the invertebrate pathogen *Metarhizium anisopliae* JEF-290 was reported to be useful to control the tick as an alternative to chemical acaricides, which are not easily applicable in human living areas where the tick is widely spread. In this study, we analyzed how the tick and the fungal pathogen interact at the transcriptional level. Field-collected tick nymphs were treated with JEF-290 conidia at 1 × 10^8^ conidia/ml. In the early stage of infection with 2.5% mortality, the infected ticks were subjected to RNA sequencing, and non-infected ticks and fungal masses served as controls. Fungus and tick genes were mostly up-regulated at the early stage of infection. In the gene set enrichment analysis of the infecting fungus, catabolic processes that included lipids, phospholipids, and detoxification processes, the response to oxidative stress, and toxic substances were significantly up-regulated. In this fungal up-regulation, various lipase, antioxidant enzyme, and hydrolase genes were highly transcribed. The gene set enrichment analysis of the infected tick showed that many peptide synthesis processes including translation, peptide metabolism, ribonucleotide metabolism, and energy production processes that included ATP generation and ADP metabolism were significantly up-regulated. Structurally, mitochondria and ribosome subunit genes in ticks were highly transcribed to upregulate these processes. Together these results indicate that JEF-290 initiates process that infects the tick while the tick actively defends against the fungal attack. This work provides background to improve our understanding of the early stage of fungal infection in longhorned tick.

## Introduction

*Haemaphysalis longicornis* (Acari: Ixodidae), the longhorned tick is an important vector for human disease, and known to transmit severe fever with thrombocytopenia syndrome virus (SFTSV), *Rickettsia japonica*, and *Coxiella burnetii* (Mahara, 1997). SFTSV, which belongs to the Phlebovirus genus in the family Bunyaviridae, was first reported in China in 2009, and then spread throughout Asia with reports in North America also (Denic et al., 2011; McMullan et al., 2012). SFTSV causes vomiting, diarrhea, high fever, and thrombocytopenia, and the mortality ranges from 2% to as high as 30% (Gai et al., 2012). Previous studies confirmed that infection occurs person to person through blood contact or bodily secretions from infected patients (Liu et al., 2012). The longhorned tick is also an important pest for livestock such as deer, sheep, and cattle. In New Zealand and Australia, the tick can reduce cattle production by 25% (Heath, 2016). This tick also mediates cattle diseases from Theileria species, which are threats to the livestock industry (Hammer et al., 2015).

The longhorned tick is a three-host ixodid tick, which takes blood meals on three different hosts at each developmental stage until engorged. The engorged adult females lay more than 2,000 eggs in the late spring and early summer. The eggs hatch into six-legged larvae in late summer and early autumn. The ticks overwinter at the nymphal stage, and become active in the following spring. The ticks suck blood for 5–7 days, and they can survive for many month without blood meal at all stages (Heath, 2016). The longhorned tick consists of a diploid and triploid population which are bisexual and obligatory parthenogenetic, respectively, and an aneuploidy population is capable of both bisexual and parthenogenetic reproduction which make longhorned tick a very interesting case of tick cytogenetics and reproduction (Oliver, 1977).

Several management tools are available to control tick (Lee et al., 2015), but most of them are not environmentally sound or effective. Most of the commonly used pesticides against ticks include pyrethroid and carbamate. Pyrethroid insecticides have high insecticidal activity but have a negative impact on the environment. In addition, mite resistance to pyrethroid-based acaricides has been recently reported (Hernandez et al., 2018). Carbamate insecticides are also highly toxic to beneficial insects, such as bees, so their use is limited. ticks inhabit parks, mountains, and lakesides, and it is difficult to spray acaricides near areas of human habitation. For this reason, various methods using biological control agents have recently been studied. Cymbopogon citratus essential oil has a relatively high mortality rate in longhorned ticks (Agwunobi et al., 2020). Another report showed that the antlion Euroleon coreaus could become a new predator for the ticks (Nwanade et al., 2020). However, control methods using predators and plant extracts have disadvantages as they are difficult to apply in large areas and rarely show high control activity (George et al., 2004; Quadros et al., 2020).

Entomopathogenic fungi may be an alternative strategy to control ticks. This fungal group is a facultative pathogen to arthropods, causes pathogenicity specifically to insects, mites and ticks, and has been used to control various pests (Charnley and Collins, 2007; Fernandes et al., 2012). Although studies on entomopathogenic fungi–mediated control of tick are still lacking, the possible effects of entomopathogenic fungi on other ticks have been shown. *Dermacentor variabilis*, *Ixodes scapularis*, and *Rhipicephalus sanguineus* showed relatively high susceptibility when Beauveria bassiana or *Metarhizium anisopliae* was applied to target ticks (Kirkland et al., 2004). In addition, entomopathogenic fungi have high virulence against engorged females and tick eggs (Perinotto et al., 2012). Recently, studies have shown that selected B. bassiana strains show high virulence against longhorned tick, and confirmed the characteristics of the strains (Zhendong et al., 2019). However, the biological control of longhorned tick using entomopathogenic fungi is unknown.

A previous study of transcriptome analysis of small planthopper, *Laodephax striatellus* has provided the information about changes of gene expression pattern upon chemical pesticide-treatment, and demonstrated that suppression of the upregulated genes synergistically improved the insecticidal effect of the pesticide (Fang et al., 2020). In considering the use of microbials on targeting pests, it is also important to understand how the two organisms respond to each other at transcriptional level because it will provide information of pathogenicity and immunity related genes which might help study of putative virulence-modulating genes. Many studies have used transcriptome sequencing of ticks to determine their defense mechanisms when parasitize their hosts (Charrier et al., 2018; Araujo et al., 2019). In addition, tick-borne diseases have been studied at the transcriptional level. In a study in Australia, RNA sequencing was conducted to study the diseases caused by various ticks (Harvey et al., 2019). In Europe, various studies have been conducted to identify diseases that ticks can carry (Vayssier-Taussat et al., 2013; Pettersson et al., 2017). However, little research has been done on the defense mechanisms in ticks during fungal infection and the fungal mechanisms during tick infection.

In this study, the entire genome of JEF-290 was sequenced by PacBio sequencing technology, application of *M. anisopliae* JEF-290 for the control of longhorned tick was evaluated, and the interaction between tick and fungal pathogen at the early stage of infection was investigated by using transcriptome *analyses*. To compare the gene expression level of JEF-290 and longhorned tick under infection, differently expressed genes (DEGs), gene ontology (GO), and gene set enrichment *analyses* were conducted, and the results were analyzed to identify potentially important tick genes for defense and fungal genes for infection. This study will help clarify and modulate how the longhorned tick and JEF-290 respond to each other at the onset of fungal infection.

## Materials and Methods

### Fungal Isolate

*M. anisopliae* JEF-290 was obtained from the Insect Microbiology and Biotechnology Laboratory (IMBL), Jeonbuk National University, South Korea. The fungal isolate was grown on quarter strength Sabouraud dextrose agar (1/4SDA; Difco, United States) in the dark at 25 ± 1°C and stored in 20% (v/v) glycerol at −80°C. This isolate has high acaricidal activity against the longhorned tick (Lee et al., 2019).

### Fungal Whole Genome Sequencing

For whole genome sequencing of *M. anisopliae* JEF-290 at Macrogen^1^ (Macrogen Inc., Seoul, South Korea), genomic DNA and RNA were extracted from 7-day-old fungal mycelia. DNA quantity was assessed by using Victor 3 fluorometry (Perkin-Elmer) with Pico-green^®^ fluorescent nucleic acid stain (Thermo Fisher Scientific). To assess DNA quality, gel electrophoresis was performed. The concentration of genomic DNA was measured using a Nano Drop spectrophotometer (Thermo Fisher Scientific) and a Qubit fluorometer (Life Technology). For PacBio RS sequencing, 8 μg of input genomic DNA was used for 20 kb library preparation. For gDNA with a size range less than 17 Kb, the Agilent 2100 Bioanalyzer system (Agilent Technologies, Palo Alto, Cambridge, United States) was used to determine the actual size distribution. Genomic DNA was sheared with g-TUBE (Covaris Inc., Woburn, MA, United States) and purified using AMPure PB magnetic beads (Beckman Coulter Inc., Brea, CA, United States). The gDNA concentration was measured using both a Nano Drop spectrophotometer and a Qubit fluorometer, and approximately 200 ng μl^–1^ of gDNA was run on a field-inversion gel. A total of 10 μl of library was prepared using the PacBio DNA Template Prep Kit 1.0 (for 3∼10 Kb). SMRT bell templates were annealed using the PacBio DNA/Polymerase Binding Kit P6. The PacBio DNA Sequencing Kit 4.0 and eight SMRT cells were used for sequencing. Subsequent steps were based on the PacBio Sample Net-Shared Protocol^2^. The genome sequences of entomopathogenic isolates such as *M. anisopliae, M. rileyi*, *M. acridum*, and *M. robertsii* were subjected to orthologous analysis. Orthologous and paralogous gene clustering analysis was performed using OrthoMCL (v.2.0.3). Data were analyzed in blastp (v2.2.25 +) (E-value 1e-5), and sequences with less than 10 amino acids or stop codon ratios exceeding 20% were excluded.

### Longhorned Tick

Wild populations of tick nymphs were collected several times from a grassland field near a rural community in Seongnam City, Korea using a carbon dioxide trapping method (Miles, 1968) from April until July in 2017 and 2018. Dry ice (2.5 kg) was placed into traps (36 × 40 cm) and traps were left in the grass field for 7 days. The collected ticks were identified based on a tick handling manual and training handbook (Yamaguti et al., 1971). Because the collected ticks consisted of approximately 95 percent of longhorned tick and less than five percent of *H. flava*, the larvae which are difficult to identify morphologically were removed from the collected colony, and only the nymphs were subjected to identification to collect ticks.

### RNA Extraction

Seventy microliter of conidial suspension of *M. anisopliae* JEF-290 (1 × 10^8^ conidia/ml) was spread on a 1/4SDA plate, and cultured at 27°C for 14 days to harvest conidia by vortexing from a sporulated agar disc (5 mm diameter) in 1.0 ml of 0.03% siloxane solution for 60 sec. The concentration of newly harvested conidia was adjusted to 1 × 10^8^ conidia/ml. Approximately 180 nymphs were dipped into the conidial suspension for approximately 30 sec, placed on a filter paper-layered petri dish, and incubated at 27°C. The ticks were collected when the tick mortality rate reached 3.0%.

Longhorned tick RNA was extracted from the infected 180 nymphs, and same number of non-infected nymphs. On the nitrocellulose membrane liner covering 1/4SDA culture petri-dishes, JEF-290 was inoculated as described above. Five days after inoculation, actively proliferating mycelia and conidia were harvested for RNA extraction as controls. Total RNAs of these samples were extracted with TRIzol reagent (Invitrogen Life Technologies, CA, United States) following the manufacturer’s instructions. RNA purity and integrity were quantified by an ASP-2680 spectrophotometer (ACTGene, Piscataway, NJ, United States) and an Agilent 2100 Bionalyzer system.

### RNA Sequencing and *de novo* Assembly

Libraries of infected tick, non-infected tick and non-infecting fungus were made using the Truseq RNA kit (Illumina, San Diego, CA, United States) following the manufacturer’s protocol at Macrogen.

The first step was to purify mRNA containing poly-A using poly-T oligo-attached magnetic beads and fragment the samples into small pieces at elevated temperature with divalent cations. The cleaved RNA fragments were reverse-transcribed into first strand cDNA with random primers.

Ampure XP beads were used in the second strand reaction mix to generate double-stranded cDNA by removing the RNA template and synthesizing the replacement strand. The 3′ overhang was removed, and polymerase was used to fill the 5′ overhang using an End Repair (ERP) mix. The adapter was then ligated to the fragment by attaching the “A” nucleotide to the 3′ end of the blunt fragment and giving a core reaction single “T” nucleotide at the 3′ end of the adapter. Multiple indexing adapters were ligated to the ends of the double-stranded cDNA and then enriched by PCR to create DNA library templates. In each isolate, infecting and non-infecting samples were sequenced in parallel using an Illumina HiSeq 2000 sequencer with a read length of 101 bp. The read quality was verified by fastQC v.0.11.8 (Andrews, 2010) and the quality was filtered to remove low quality sequences with a Phred score of 30 or less using NGS QC Toolkit v.2.3.3 (Patel and Jain, 2012). For efficient and robust *de novo* reconstruction of transcriptomes, Trinity (ver 2.8.3) was used^3^. TransDecoder (version 5.5.0) was used to identify candidate coding regions within the transcription sequence. Contigs with more than 90% of sequence identities were clustered using cd-hit-est v.4.8.1 (Fu et al., 2012) to remove isoforms, and the *in silico* cDNA library was constructed.

### Differentially Expressed Gene and Gene Ontology Analyses

To quantify transcript abundances, Kallisto (ver 0.45.0) was used to build an index form from the fasta form of target sequences and non-infecting and infecting libraries were compared. Transcripts per million (TPM) of non-infecting and infecting samples was calculated. Raw signals were normalized using a log_2_-based transformation. Fold-change statistical tests were performed and log_2_|FC| $\underset{=}{>}$2 was defined as statistically significant differential expression. Contigs were blasted using the Blast2Go program with a local blast. The statistical significance threshold was 1.0E-10 and the number of blast hits was set to one. GO analysis of up- and down-regulated contigs was performed using InterPro (online) in the Blast2Go program. The public EMBL-EBI database was used to scan sequences against InterPro’s signatures. Up- and down-regulated contigs were annotated at GO level 2. In addition, using the Blast2Go program, the tick contigs were mapped to the local immune database that based on ImmunoDB^4^ was prepared by downloading immune genes of *Drosophila melanogaster* from NCBI to analyze the up-regulation of immune-related contigs in the infected tick.

### Validation of RNA-Sequencing

Six and five randomly selected genes of tick and *M. anisopliae* JEF-290 were validated using qRT-PCR, respectively. The RNA samples from infected tick and non-infected tick, and non-infecting JEF-290 were subjected to reverse transcription (RT) using an AccuPower^®^ RT PreMix (Bioneer, Daejeon, South Korea) with oligo (dT) 15 primer (Promega, MI, United States). The qRT-PCR primers were designed at SnapDragon^5^. qRT-PCR was performed using Thunderbird^®^ SYBR^®^ qPCR mix (QPS-201, TOYOBO, Japan) in a 96-well Bio-Rad CFX96 Real-Time PCR System (Bio-Rad, United States). PCR conditions were as follows: denaturation for 1 min at 95°C, and then 40 cycles of 15 s at 95°C, 1 min at 60°C followed by melting, increased 0.5°C per 5 s started from 65 to 95°C. The total RNA without reverse transcription was used as a negative control, and different *actin* genes from tick and JEF-290 were independently used as an internal control to normalize relative expression level. In this validation, other housekeeping genes were tried but they worked well, so *actin* genes were finally used. All experiments were performed in triplicate. △Ct (threshold cycle) was calculated as (Ct value of up-regulated genes)–(Ct value of *actin*) and subjected to the calculation of fold change value (2^−ΔΔCt^).

### Gene Set Enrichment Analysis

DEG contigs of infecting *M. anisopliae* JEF-290 and infected longhorned tick with more than twofold change difference were subjected to GO enrichment analysis to identify the significantly involved functional gene groups in infection and defense. Blastn was performed with an *E*-value of 1.0E-10 and non-blasted and non-GO ID contigs were removed. As a reference, *Ixodes scapularis* and *Beauveria bassiana* were used. Functional genes set enrichment was performed using the g:Profiler web server^6^. The related *p*-value was corrected for multiple testing using the Benjamini-Hochberg False Discovery Rate (FDR) procedure with a threshold of 0.05.

## Results

### Whole Genome of *M. anisopliae* JEF-290

As a result of whole genome sequencing of *M. anisopliae* JEF-290, a total of 11,868,389,082 bases were identified with N50 of 26,851 bp (Supplementary Table 1). The number of reads was 746,811 and the mean subread length was 15.9 Kb. A total of 18 contigs were assembled and the whole genome size was analyzed to be 42.85 Mb with 13,634 coding genes, which was larger than other *Metarhizium* species (Table 1). Comparison of the orthologs of the six different genus of entomopathogenic fungi revealed that all strains shared 5,320 genes, and JEF-290, M. robertstii, M. riley, and *M. acridum* shared 698 genes (Supplementary Figure 1).

**TABLE 1:** Genome features of *M*. *anisopliae* JEF-290 and other entomopathogenic fungi.

**Features**	***Ma* JEF-290**	***M. anisopliae***	***M. robertsii***	***M. acridum***	***M. rileyi***	***Bb* JEF-007**	***C. militaris***
Size (Mb)	42.8	38.5	39	38.1	32.0	36.5	32.2
Coverage (fold)	237X	98.3X	100X	107X	107.3X	105.1X	147X
Scaffold No. (>1 kb)	18	74	176	241	389	39	13
Scaffold N50 (Mb)	6.25	2.04	1.96	0.33	0.89	3.12	4.55
% G + C content	50.9	50.7	51.5	50	49.3	48	51.4
% G + C in coding gene	54.3	54.5	54.4	54.1	54.3	57.1	58.6
% Repeat rate	1.32	0.89	0.98	1.52	1.51	1.71	3.04
Protein-coding genes	13654	10891	10,582	9,849	8764	10,857	9,684
Protein families (protein no.)	4848 (8310)	6401 (8554)	2,797^b^ (7,556)	2,746^b^ (6,948)	6261 (6852)	1,284^a^ (4,282)	2,736^*b*^ (6,725)
Gene density (gene per Mb)	319	283	271	259	274	297	301
Exons per gene	2.2	2.6	2.8	2.7	2.8	2.3	3
% Secreted proteins	16.2	19.5	17.6	15.1	19.5	18.9	16.2
tRNA	274	152	141	122	113	140	136
NCBI Accession No.	PRJNA530366	AZNF00000000.1	ADNJ00000000	ADNI00000000	AZHC00000000.1	PRJNA352877	AEVU00000000

*^*a*^MAKER analysis. ^*b*^InterProScan analysis.*

### Infection of *M. anisopliae* JEF-290 to Longhorned Tick

**FIGURE 1:**
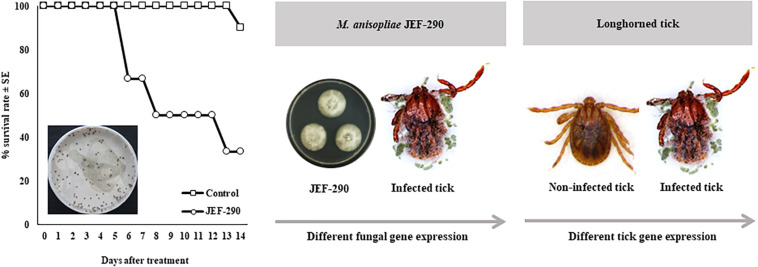
Virulence of *M. anisopliae* JEF-290 against nymphs of longhorn ticks in laboratory conditions.

Under laboratory conditions, application of *M. anisopliae* JEF-290 to nymphs resulted in high control efficacy and mycosis (Figure 1). The survival rate of nymphs in the non-treated control was 90.0% at 15 days post-application, but JEF-290 treatment resulted in approximately 66.7% mortality at 15 days and >90% of mortality at 30 days [*F*_(2, 143)_ = 241.9, *p* < 0.001]. Fungal mycosis was observed on the cadavers of infected nymphs.

### Comparative Differentially Expressed Genes of *M. anisopliae* JEF-290 and Longhorned Tick

**FIGURE 2:**
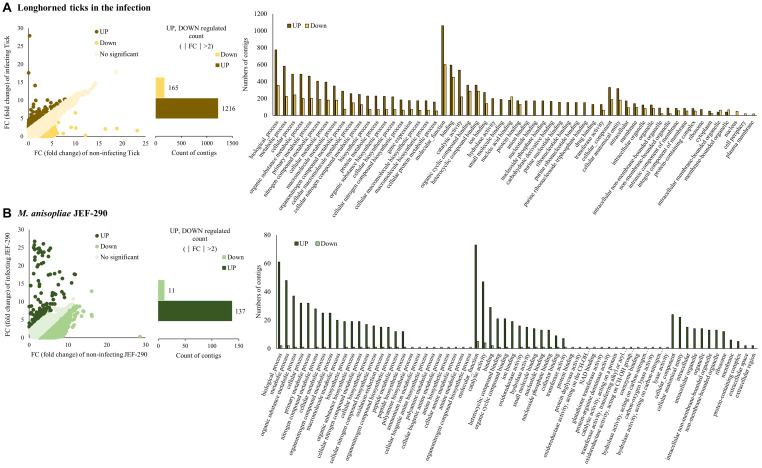
Differentially expressed genes (DEG) of **(A)** longhorned tick and **(B)** *M. anisopliae* JEF-290 at early stage of infection. Longhorned ticks were exposed to 14 days old fungus cultured plates for 14 days. As a non-infecting control, 5 days cultured fungus was used for RNA extraction. In each isolate, the numbers of up- and down-regulated contigs (|fold change| > 2) were analyzed and GO *analyses* of DEGs were conducted using Blast2Go program.

From analyzing the RNA sequences, 8.767 Gb (longhorned tick), 8.549 Gb (JEF-290) and 11.224 Gb (infected tick) of raw sequences were identified (Supplementary Table 2). Filtering and *de novo* assembly were performed based on the raw data above to obtain a sequence of 27.2, 16.5, and 28.9 Mb, respectively. The assembled contigs of longhorned tick, JEF-290 and infected tick were clustered with more than 90% identity using the CD-hit-est program, and the numbers of clustered contigs were 33,099 (longhorned tick), 15,044 (JEF-290), and 36,292 (infected tick), respectively. The N50 values of the samples were 1101 b (longhorned tick), 1554 b (JEF-290), and 1056 b (infected tick). In the DEG analysis, relatively large numbers of tick and fungal genes were up-regulated when the tick was infected by the fungus (Figure 2). Among the tick genes, 1216 genes were up-regulated and 165 genes were down-regulated (|FC| > 2). Among the fungal genes, 137 genes were up-regulated and 11 genes were down-regulated (|FC| > 2) (Supplementary Table 3). qRT-PCR was performed on the randomly selected genes (Supplementary Table 4), and the results confirmed that gene expression levels determined by qRT-PCR and RNA-seq analysis results were similar to each other although limited numbers of internal control genes were used (Supplementary Figure 2).

### Gene Ontology of Differentially Expressed Genes

In the GO analysis of the infected tick, fungal GO terms were almost up-regulated, but GO terms of longhorned tick were mostly up-regulated with partial down-regulation (Figure 2). In the GO of longhorned tick genes, 1,381 DEGs were classified in the following three GO terms: biological process, cellular components, and molecular function (Figure 2A). Many longhorned tick contigs belonging to biological process and molecular function showed a trend that more of these contigs were up-regulated while the contigs belonging cellular component showed relatively less changes of gene expression.

The up-regulated longhorned tick GO terms (FC > 2) included 1,216 genes that included biological process (54%), molecular function (36%), and cellular component (11%), and these GO terms were mainly metabolism, catalytic activity, and bindings. The contigs annotated as being involved in metabolic process (GO:0008152), cellular process (GO:0009987), and organic substance metabolic process (GO:0071704), primary metabolic process (GO:0044238), catalytic activity (GO:0003824), hydrolase activity (GO:0016787), small molecule binding (GO:0036094), anion binding (GO:0043168), and nucleotide binding (GO:0000166) were upregulated noteworthily. The down-regulated longhorned tick GO terms (FC < 2) included 165 genes that included biological process (59%), molecular function (29%), and cellular component (11%). The contigs annotated as being involved in organic cyclic compound binding (GO:0097159), heteroclyclic compound binding (GO:1901363), nucleic acid binding (GO:0003676), and protein binding (GO:0005515) were down-regulated noteworthily.

In the GO of *M. anisopliae* JEF-290, 295 DEGs were categorized as three GO types: biological process, cellular component, and molecular function (Figure 2B). The up-regulated fungal GO terms (FC > 2) included 137 genes that included biological process (53%), molecular function (36%) and cellular component (12%). The up-regulated GO terms were mainly metabolic process and binding. The contigs annotated as being involved in organic substance metabolic process (GO:0071704), nitrogen compound metabolic process (GO:0006807), macromolecule metabolic process (GO:0043170), cellular metabolic process (GO:0044237), biosynthetic process (GO:0009058), oxidoreductase activity (GO:0016491), hydrolase activity (GO:0016787), transferase activity (GO:0016740), cellular anatomical entity (GO:0110165), and intracellular organelle (GO:0043229) were up-regulated noteworthily. The down-regulated fungal GO terms (FC < 2) included 11 genes that included biological process (43%) and molecular function (57%).

### Immune-Related Upregulated Longhorned Tick Genes

Mapping of the longhorned tick contigs to a local immune database from ImmunoDB revealed that three serpin genes were up-regulated at the early stage of fungal infection (Table 2). Other immune-related genes did not show any significant up- and down-regulations. From the mapping, two uncharacterized genes (Infect_tick_13855 and Infect_tick_25741) and three serpin genes (Infect_tick_1165, Infect_tick_1329 and Infect_tick_13414) were upregulated when the tick was infected by *M. anisopliae* JEF-290. Other immune-related genes were mapped to the database, but most of the genes were not strongly affected by the fungal infection (|FC| < 1): *D. melanogaster cactus*, *pelle* and *caspar* genes, and *peroxidasin*, *thioredoxin peroxidase*, and other serpin genes. *Serpin 28DC, 55B*, and *43Ab* genes were upregulated, but the other mapped serpin genes (*serpin 27A, 28F, 28B, 42Dc, 42De, 43Aa, 43Ad, 77Ba*, and *100A*) were not significantly changed.

**TABLE 2:** Immune-related genes of infected longhorned tick at early stage of infection.

**Contig No.**	**length**	**Immune group**	**Description**	***e*-value**	**sim mean (%)**	**log_2_FC value**
Infect_tick_20112	2557	IMDPATH	Drosophila melanogaster caspar (casp), transcript variant A, mRNA	3.44E-78	68.42	0.370982
Infect_tick_11706	2488	IMDPATH	Drosophila melanogaster I-kappaB kinase beta (IKKbeta), mRNA	5.91E-36	63.51	0.451297
Infect_tick_11636	3249	IMDPATH	Drosophila melanogaster TAK1-associated binding protein 2 (Tab2), transcript variant A, mRNA	6.65E-12	56.76	−0.12631
Infect_tick_17827	3391	IMDPATH	Drosophila melanogaster TGF-beta activated kinase 1 (Tak1), transcript variant A, mRNA	3.75E-89	81.25	−0.43068
Infect_tick_14945	5055	JAKSTATs	Drosophila melanogaster hopscotch (hop), mRNA	9.36E-43	60.36	−0.59627
Infect_tick_4516	3595	JAKSTATs	Signal-transducer and activator of transcription protein at 92E (Stat92E), transcript variant B, mRNA	4.09E-89	76.25	−0.31486
Infect_tick_11603	4388	LYSs	Uncharacterized protein (CG8492 LYS-long), mRNA	4.22E-38	79.71	0.626852
Infect_tick_13855	615	LYSs	Uncharacterized protein (CG16756), mRNA	6.33E-26	74.36	1.656939
Infect_tick_25189	641	PRDXs	Uncharacterized protein (CG15116), mRNA PRDX9	4.84E-20	62.75	0.40434
Infect_tick_25741	5000	PRDXs	Uncharacterized protein, transcript variant A (CG10211)	6.5E-113	68.94	1.184511
Infect_tick_28125	2086	PRDXs	Transcript variant B (CG4009), mRNA PRDX13	5.64E-47	62.26	0.625927
Infect_tick_10092	5336	PRDXs	Dual oxidase, transcript variant B (Duox), mRNA PRDX20	0	88.49	−0.15924
Infect_tick_19211	5126	PRDXs	Peroxidasin, transcript variant A (Pxn), mRNA PRDX15	0	75.29	0.824367
Infect_tick_34328	1010	PRDXs	Thioredoxin peroxidase 1 (Jafrac1), transcript variant A, mRNA PRDX8	1.4E-105	88.42	0.33423
Infect_tick_18570	1047	PRDXs	Thioredoxin peroxidase 2, transcript variant A (Jafrac2), mRNA PRDX7	1.3E-104	83.84	0.51408
Infect_tick_2240	848	PRDXs	Uncharacterized protein (CG12896), mRNA PRDX5	1.66E-70	68.24	0.699156
Infect_tick_9192	1017	PRDXs	Peroxiredoxin 3 (Prx3), mRNA PRDX6	3.1E-85	78.49	0.423467
Infect_tick_35967	1633	SRPNs	Necrotic (nec), mRNA	2.76E-47	53.99	0.268997
Infect_tick_18887	1195	SRPNs	Serpin 27A, transcript variant A (Spn27A), mRNA	2.68E-46	55.26	0.108819
Infect_tick_22860	1213	SRPNs	Serpin 28F (Spn28F), mRNA	1.03E-46	61.46	0.540716
Infect_tick_28060	1248	SRPNs	Serpin 28B, transcript variant A (Spn28B), mRNA	8.31E-39	52.46	0.080996
Infect_tick_13414	1792	SRPNs	Serpin 28Dc (Spn28Dc), mRNA	5.75E-20	49.18	2.346453
Infect_tick_18886	1621	SRPNs	Serpin 42Dc, transcript variant A (Spn42Dc), mRNA	2.87E-45	66.27	0.233116
Infect_tick_28061	1311	SRPNs	Serpin 42De, transcript variant A (Spn42De), mRNA	3.21E-48	55.38	0.481642
Infect_tick_12140	1294	SRPNs	Serpin 43Aa (Spn43Aa), mRNA	1.49E-43	52.7	0.455747
Infect_tick_1165	1297	SRPNs	Serpin 55B (Spn55B), mRNA	4.82E-38	55.68	1.233754
Infect_tick_1166	1992	SRPNs	Serpin 77Ba, transcript variant A (Spn77Ba), mRNA	1.56E-18	46.32	0.795475
Infect_tick_2329	2038	SRPNs	Serpin 100A (Spn100A), mRNA	1.66E-23	50	0.536968
Infect_tick_13636	1329	SRPNs	Serpin 43Ab (Spn43Ab), mRNA	8.86E-29	52.26	1.765842
Infect_tick_12140	1432	SRPNs	Serpin 43Ad (Spn43Ad), mRNA	4.96E-19	49.34	0.455747
Infect_tick_16761	1893	TOLLPATH	Drosophila melanogaster cactus (cact), transcript variant A, mRNA	2.36E-29	57.02	−0.05294
Infect_tick_33902	1931	TOLLPATH	Drosophila melanogaster pelle (pll), transcript variant A, mRNA	4.42E-62	74.47	−0.30926
Infect_tick_30771	1608	AMP	Drosomycin-like1	5E-31	100	1.56264

### Gene Ontology Enrichment Analysis

**FIGURE 3:**
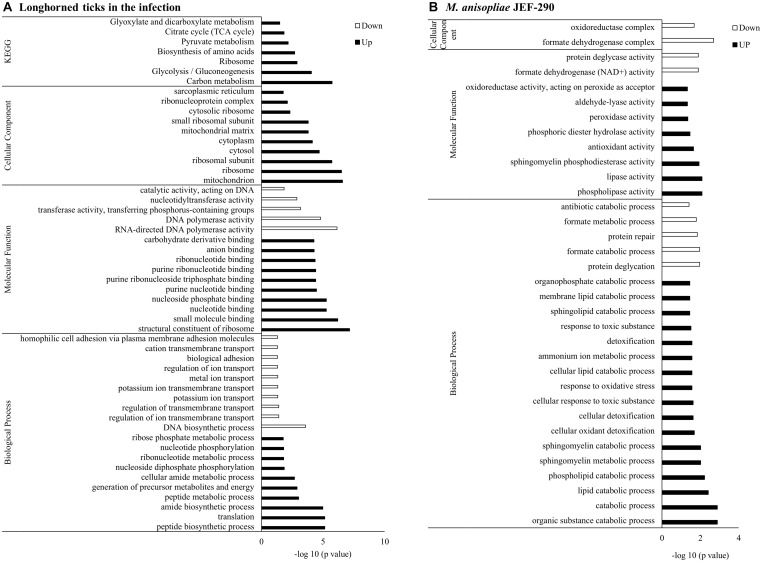
GO enrichment of longhorned tick **(A)** and *M. anisopliae* JEF-290 **(B)** DEGs at early stage of infection. Longhorned tick in the infection and *M. anisopliae* JEF-290 that showed more than ± 2 fold change difference were subjected to GO enrichment analysis using g:Profiler to investigate significant functional differences when infecting ticks and *M. anisopliae* JEF-290. The *M. anisopliae* does not provide enough gene IDs for enrichment analysis, so alternatively Beauveria bassiana with much larger gene IDs was used as a reference and the *p*-value was corrected for multiple testing using the FDR procedure with a threshold of 0.05.

**FIGURE 4:**
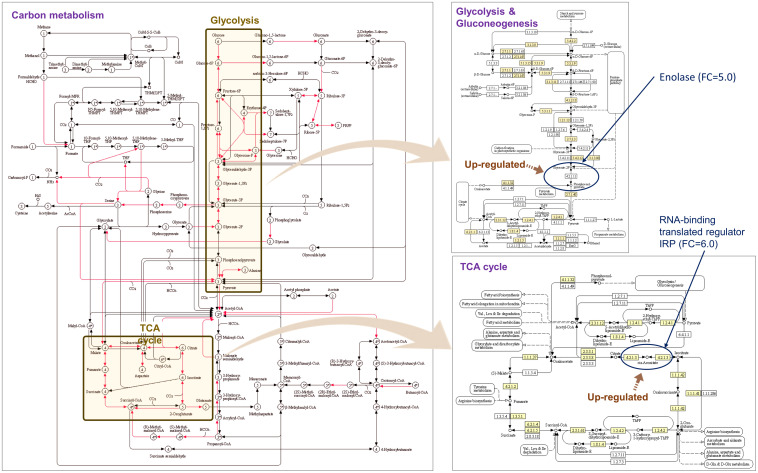
Enriched pathways of infected longhorned tick. KEGG pathway were summarized. Yellow box parts of the KEGG pathway can be involved with Glycolysis and Gluconeogenesis and TCA cycle (figure from KEGG database).

Gene set enrichment analysis of the DEGs showed that the GO terms related to peptide synthesis processes and cellular energy production of longhorned tick were over-represented while those of catabolic processes were over-represented in *M. anisopliae* JEF-290 was working on catabolic processes to degrade metabolites (Figure 3). In the infected tick, seven tick pathways and 30 tick GO terms were significantly involved in the fungal infection (Figure 3A). The major up-regulated GO terms were peptide biosynthetic process (GO:0043043), translation (GO:0006412), amide biosynthetic processes (GO:0043604), and peptide metabolic processes (GO:0006518). Up-regulated longhorned tick genes were related to the following pathways: 18 genes were related to carbon metabolism (KEGG:01200), nine genes to glycolysis/gluconeogenesis (KEGG:00010), 14 genes to ribosomes (KEGG:03010), nine genes to biosynthesis of amino acids (KEGG:01230), seven genes to pyruvate metabolism (KEGG:00620), six genes to the citrate cycle (KEGG:00020), and senven genes to glyoxylate/dicarboxylate metabolism (KEGG:00630). In the enriched pathways of infected tick, the RNA-binding translation regulator IRP (FC = 6.0) and enolase (FC = 5.) were highly up-regulated in the TCA cycle and glycolysis pathways, respectively (Figure 4). The infected tick also significantly upregulated peptide production and energy production pathways. The 26 GO terms in JEF-290 were significantly involved in the fungal infection (Figure 3B). The major up-regulated GO terms were lipid catabolic processes, phospholipid catabolic processes, cellular oxidant detoxification, cellular detoxification, and response to oxidative stress. JEF-290 significantly up-regulated lipid degradation and detoxification against the host defense (Figure 3B).

## Discussion

### Representative Features of *M. anisopliae* JEF-290 Genome

In this RNA-sequencing study, the whole genome of *M. anisopliae* was very useful for fungal gene characterization. The genome size of *M. anisopliae* JEF-290 is 42.8 Mb, which is larger than those of other *Metarhizium* species (32∼39 Mb). JEF-290 was found to contain 99 specific genes those were not found in the other analyzed species. Many JEF-290 genes were shared with other *Metarhizium* species and other genus of entomopathogenic fungi, however, this isolate has some unique genes which were found to be involved in a variety of fungal life activities. Alpha-beta hydrolase is a structure-related enzyme with various catalytic functions (Holmquist, 2000). In general, the alpha-beta hydrolase enzyme is involved in the breakdown of cellular metabolites (Hotelier et al., 2004). The unique ankyrin repeat protein is involved in protein-protein interactions. It exists in most organisms and is involved in cell cycle control, transcriptional regulation, cell signaling, development, differentiation, apoptosis, cellular scaffolding, and plant defense (Li et al., 2006). The ubiquitin-protein ligase in JEF-290 is actively involved in the protein conversion mechanisms that play an important role in the regulation of various cellular functions. Ubiquitin-protein ligase is also involved in activating toll-like receptors (Chuang and Ulevitch, 2004). The ribonuclease H-like (RNHL) superfamily group is involved in nucleic acid-related biological processes including replication, homologous recombination, DNA repair, transposition, and RNA interference (Majorek et al., 2014). Transposase-like proteins are generally known as mobile genetic units and have the potential to be mobilized by stress.

### Host and Fungal Gene Expression at the Onset of Infection

In the early infection stage, JEF-290 possibly initiates infection steps while the longhorned tick recognizes the fungal infection, and is attempting to defend against infection. However, once the infected hosts died, the fungus becomes saprophytic. Comparative transcriptome analysis of the early stage of fungal infection may provide more informative interactions between the tick and entomopathogenic fungus, including fungal initiation of infection and host response to the fungal infection. On the other hand, it would not be possible to observe much of an interactive response of ticks against fungal infection in the late infection stage showing more than 90% of host mortality, therefore RNA-sequencing would not provide meaningful information of the interaction.

In the analysis of longhorned tick and JEF-290 transcripts, many up-regulated contigs were found in both pathogen and host at the early infection stage. Our results showed that the longhorned tick recognizes JEF-290 infection, and increases the expression of defense and immune related contigs. However, the contigs which are supposed to transcribe very short mRNAs, such as antimicrobial peptide genes were not be able to be analyzed properly because the standard sequencing library preparation protocol does not target such short mRNAs. Therefore the library preparation protocol need to be modified for further study of low molecules. Additionally, cloning and sequencing of the transcripts is indispensable for reliable validation of Illumina sequencing results and further functional study using qPCR.

From the mapping of longhorned tick contigs to the immunoDB, three serpin genes were identified as up-regulated. Serpin is a well-known family of serine protease inhibitors that are typically around 45 kDa. Serpin regulates the arthropod immune reaction through the inhibition of serine protease reactions that initiate the melanization cascade and antimicrobial peptide production (Reichhart et al., 2011; Meekins et al., 2016), therefore the insects, ticks, and mites have serpin genes in their genomes (Mulenga et al., 2009; Rodriguez-Valle et al., 2015; Rider et al., 2015). Forty five serpin genes were found in the blacklegged tick *Ixodes scapularis*, 22 in the cattle tick Rhipicephalus microplus, and only 10 in the scabies mite Sarcoptes scabiei. Some immune-responsive serpins function at the early stage of microbial infection, and are highly up-regulated in the response to microbial infection or physical injury (Meekins et al., 2016). Several studies on arthropod serpins have been reported, but still an interactive response between arthropods and entomopathogenic fungi did not fully explain the up- and down-regulation of serpin gene-expression.

### Infection-Specific Gene Expressions of the Fungus and Tick

In the gene set enrichment analysis of longhorned tick, pathways related to protein synthesis and energy production such as translation, peptide metabolism, ribonucleotide metabolism, energy production process including ATP generation, and ADP metabolism were up-regulated. These results suggest that the longhorned tick actively secreted protective substances against the invasion of JEF-290. Of the several pathways obtained from the enrichment analysis of longhorned tick, the RNA-binding translational regulator IRP gene was significantly involved in the management of fungal challenge in most of the significant pathways. When the longhorned tick was infected by the fungus, this gene was highly up-regulated (FC = 6.0). RNA binding proteins (RBP) regulate the translation of mRNA by interacting with 5’ and 3’ untranslated regions (UTR) of mRNA (Harvey et al., 2018). RBP is involved in the assembly of mRNA to the ribosomes and regulates protein synthesis or alternatively suppresses translation (Moore and Lindern, 2018). Among the RBPs, iron regulatory protein (IRP) binds to RNA stem-loops of UTR to manipulate mRNA translation (Zhang et al., 2002) and maintains homeostasis of iron concentrations in mammalians including in response to blood-sucking ticks (Anderson et al., 2012). IRP regulates not only iron homeostasis-related mRNAs, but also other mRNAs such as mitochondrial acotinase (ACO2) mRNA, which has an iron responsive element (IRE) and encodes a protein involved in the tricarboxylic acid cycle (TCA) catalyzing citrate to isocitrate (Wang and Pantopoulos, 2011). Acotinase has a 4Fe-4S iron-sulfur cluster as an IRE that directly interacts with the IRP. We speculate that when longhorned tick is infected by JEF-290, IRP possibly binds to the IRE of acotinase mRNA of tick for gene regulation, however, clear evidence remains to be demonstrated.

In the gene set enrichment analysis of JEF-290, fungal pathways such as catabolic processes including lipid, phospholipid, sphingomycelin, and sphingolipid pathways, and detoxification processes including response to oxidative stress and toxic substances were to be up-regulated. We speculate that JEF-290 easily degrades insect cuticles during penetration of the host insect body. In addition, degrading host defense substances possibly makes the fungal invasion much easier. In entomopathogenic fungi, the cytochrome P450 (CYP450) gene encodes a protein that is involved in the degradation of lipid and wax layers of arthropods (Shin et al., 2020). In RNA-sequencing of western flower thrips infected with B. bassiana ERL836 or JEF-007, two different CYP genes (CYP539B1 and CYP655C1) were identified in the infecting fungal isolates (Kim et al., 2020). CYP52 and CYP53 degrade insect lipid layers and assimilate cuticular hydrocarbons (Huarte-Bonnet et al., 2018). In fungal detoxification, most substrate-degrading fungi contain a detoxification system, named the xenomic network, which includes the cytochrome P450 (CYP450) family (Phase I) and glutathione transferase (GST) family (Phase II) (Schrand et al., 2010; Meux et al., 2011). These proteins are involved in the modification of exogenous compounds through oxidative reaction and conjugation, respectively. As discussed above, entomopathogenic fungi produce various CYPs, and they are involved in both fatty acid degradation and fungal detoxification against the arthropod immune response perhaps to work as a cuticle penetrator and defender against arthropods. More studies need to be conducted to explore this potential mechanism.

**FIGURE 5:**
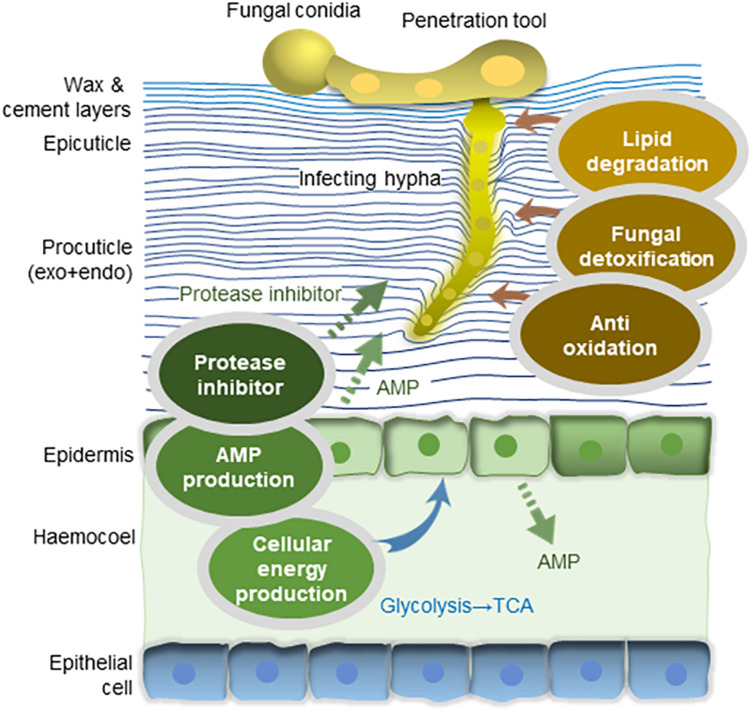
Interaction of *M. anisopliae* JEF-290 and longhorned tick at early stage of fungal infection. Genes from the longhorned tick and JEF-290 fungus were mostly up-regulated at the early stage of fungal infection. Our results indicate that JEF-290 initiates infection of the longhorned tick by degrading host cuticles with a detoxification tool, while the longhorned tick was actively defending against the fungal attack by producing a large amount of energy by inducing catabolism processes (original illustration).

In summary, genes from the longhorned tick and JEF-290 fungus were mostly up-regulated at the early stage of fungal infection. Our results indicated that JEF-290 initiates infection of the longhorned tick by degrading host cuticles with a detoxification tool while the longhorned tick was actively defending against the fungal attack by producing a large amount of energy by inducing catabolism processes (Figure 5). These findings may provide a strong background to understand the early stage of fungal infection of the longhorned tick. Comparative transcriptome analysis needs to be combined with genetic variation study for better understanding of the tick and fungus interaction, and searching for virulence-related marker genes of both pathogen and host.

## Data Availability Statement

The datasets presented in this study can be found in online repositories. The names of the repository/repositories and accession number(s) can be found in the article/Supplementary Material.

## Author Contributions

ML designed this work and analyzed RNA-sequencing raw data. JK and WK analyzed the RNA-sequencing data. ML and SP extracted DNA and RNA from the samples. D-HL collected longhorned tick from fields and identified. JK designed the whole experiments and wrote the manuscript. All authors approved the submission of this manuscript.

## Conflict of Interest

The authors declare that the research was conducted in the absence of any commercial or financial relationships that could be construed as a potential conflict of interest.

## Footnotes

1 www.macrogen.com

2 http://pacificbiosciences.com/

3 https://github.com/trinityrnaseq/

4 http://cegg.unige.ch/Insecta/immunodb

5 http:/www.flyrnai.org/snapdragon

6 http://bitt.cs.ut.ee/gprofiler/

## Supplementary Material

Supplementary Figure 1 Reciprocal analysis of *M. anisopliae* JEF-290 and other entomopathogenic fungi.

Supplementary Figure 2 Validation of RNA-sequencing data using qRT-PCR.

Supplementary Table 1 Raw data and assembly of *M. anisopliae* JEF-290 contigs for whole genome prediction. The sequencing of whole genomes using Pac Bio RSII technology with error correction.

Supplementary Table 2 Summary of the longhorned tick, *M. anisopliae* JEF-290 and infected longhorned tick *in silico* cDNA library.

Supplementary Table 3 TPM values and fold changes of JEF-290 and longhorned tick contigs.

Supplementary Table 4 Primers used in qRT-PCR for validation of RNA-sequencing.
