# Supplementary material for: Interactive Gene Expression Between Metarhizium anisopliae JEF-290 and Longhorned Tick Haemaphysalis longicornis at Early Stage of Infection
Source: Front Physiol. 2021 May 19;12:643389. doi: 10.3389/fphys.2021.643389 (PMC8170561; doi:10.3389/fphys.2021.643389)
Supplement: Supplementary Figure 1 — Reciprocal analysis of M. anisopliae JEF-290 and other entomopathogenic fungi. [file Data_Sheet_1.PDF]

Table S1. Raw data and assembly of *M. anisopliae* JEF-290 contigs for whole genome prediction.

Mean Subread length	Total Number of Bases	N50	Number of Reads
15,892	11,868,389,082	26,851	746,811

Number of contigs	Total Length[1]	N50[2]	Max Length[3]	Min Length[4]	Avg Length[5]
18	42,848,098	6,254,943	10,418,148	9,890	2,380,449

[1]Total length: the total length of contigs
[2]N50: 50% of all bases come from contigs longer than this value
[3]Max length: the length of maximum contig
[4]Min length: the length of minimum contig
[5]Avg length: the average length of contigs assembled
[6]Depth: the number of reads that overlap each contig

Contig Name	Length (bp)	GC %	Depth
contig1	10,418,148	51.0	235
contig2	7,896,085	51.7	237
contig3	6,254,943	51.3	239
contig4	5,196,790	50.5	235
contig5	4,335,593	51.3	233
contig6	3,178,362	51.7	241
contig7	2,749,252	50.3	233
contig8	1,222,952	49.6	234
contig9	959,584	48.1	235
contig10	160,537	28.4	559
contig11	101,514	28.3	70
contig12	92,948	49.9	193
contig13	75,461	45.6	225
contig14	70,228	51.6	507
contig15	54,518	51.6	620
contig16	38,119	51.5	26
contig17	33,174	51.8	30
contig18	9,890	51.5	12
Total	42,848,098	50.90	237

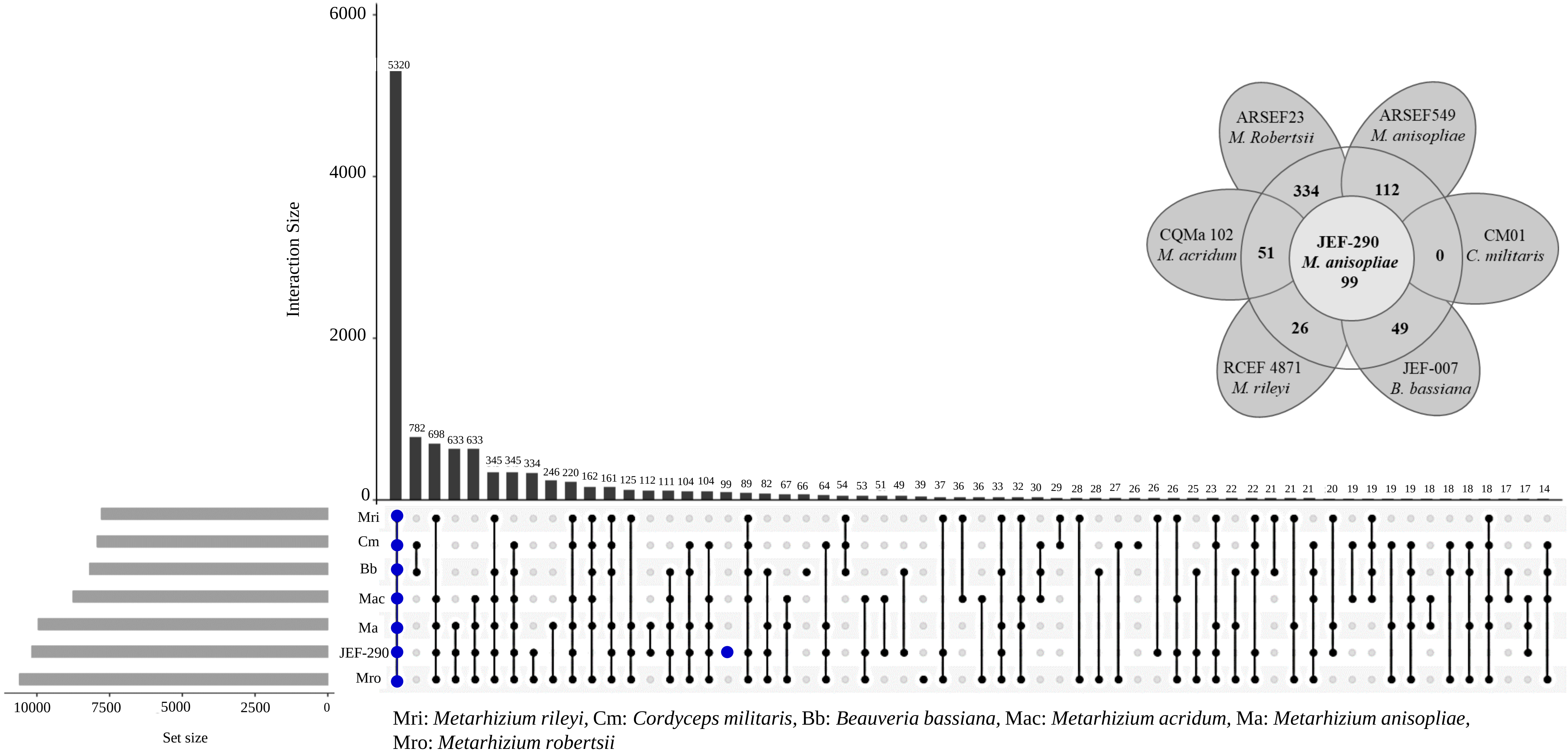

Mri: *Metarhizium rileyi*, Cm: *Cordyceps militaris*, Bb: *Beauveria bassiana*, Mac: *Metarhizium acridum*, Ma: *Metarhizium anisopliae*, Mro: *Metarhizium robertsii*

Figure S1. Reciprocal analysis of *M. anisopliae* JEF-290 and other entomopathogenic fungi.

Table S2. Summary of the longhorned tick, *M. anisopliae* JEF-290 and infected longhorned tick in silico cDNA library

Category	Sample	*H.longicornis*	*M. anisopliae*_JEF290	JEF290_Infected tick
Raw Data using Illumina sequencer	Total length of sequence (bp)	8,767,901,910	8,549,865,130	11,224,399,872
	**Total number of contig**	**86,810,910**	**84,652,130**	**111,132,672**
	GC%	52.30	49.28	51.51
in silco cDNA library	Total length of sequence (bp)	27,200,409	16,527,099	28,960,482
	Total number of contig	33,099	15,044	36,292
	N25 statistics (sequences/bp)	2,107/2,046	1,129/2,478	2,397/1,929
	**N50 statistics (sequences/bp)**	**6,734/1,101**	**3,277/1,554**	**7,575/1,056**
	N75 statistics (sequences/bp)	15,426/549	6,734/894	17,150/531
	GC%	57.48	54.18	56.84

Table S4. Primers used in qRT-PCR for validation of RNA-sequencing

Name	Sequences (5′-3′)		Target gene
	Forward primer	Reverse primer	
Infect_tick_35351	AACCCAGCACTGATGGTAGG	CAGTTGGTCACAATTCCGTG	Actin (JEF-290)
Infect_tick_1999	TACGAATGCAGAGTTGGCTG	ATGTTCGCTATGTCGTTCCC	Actin (Longhorned tick)
Infect_tick_1447	TGAGTCGGTTGCAAAGACAG	TGCTGCTCGATAGTGACGAC	S-adenosylmethionine synthetase
Infect_tick_18816	CGACGACTGCACCAAGTACA	TTGGTCAGCTTGTCGAAGTG	Salivary mucin
Infect_tick_13357	GTGAAGAAGTCCATCGAGGC	GTTGACGACCTTGGTGTCCT	Histone H1
Infect_tick_1690	GGGTGGATCAGGTTAGGGAT	AGATTGTAGCGCAGGCTCAT	Serine proteinase inhibitor serpin-3
Infect_tick_3711	TACCGAAAGAGATGGATGGC	TCCAGCGCACTCTTCTACCT	Unknown
Infect_tick_33372	TGAAGTCACCACTGAGGCTG	TGATATTTCTTGCGGCCTTT	Kettin
Infect_tick_757	GAGTACGCACTCAAGCAGCA	CGTCGTCCACTTCAGAGATG	Unknown
Infect_tick_23294	CTTGTGATATTCCGGGCATC	ATCCTTGGGCACATCATAGC	Hypothetical protein location
Infect_tick_30510	ATTACATCGGCGAGGACAAC	AGAGTAAGGCGCCAACTTGA	Fucose-specific lectin
Infect_tick_4115	CTGGAACTTCGCTTGATGCT	TTGTTTTCCTCACCGTAGGC	Formate dehydrogenase
Infect_tick_12361	GCCAGCGCTACTATCTCACC	GACAATCTCGTACCCGTCGT	Protein/nucleic acid deglycase HchA

(a) Longhorned ticks in the infection.

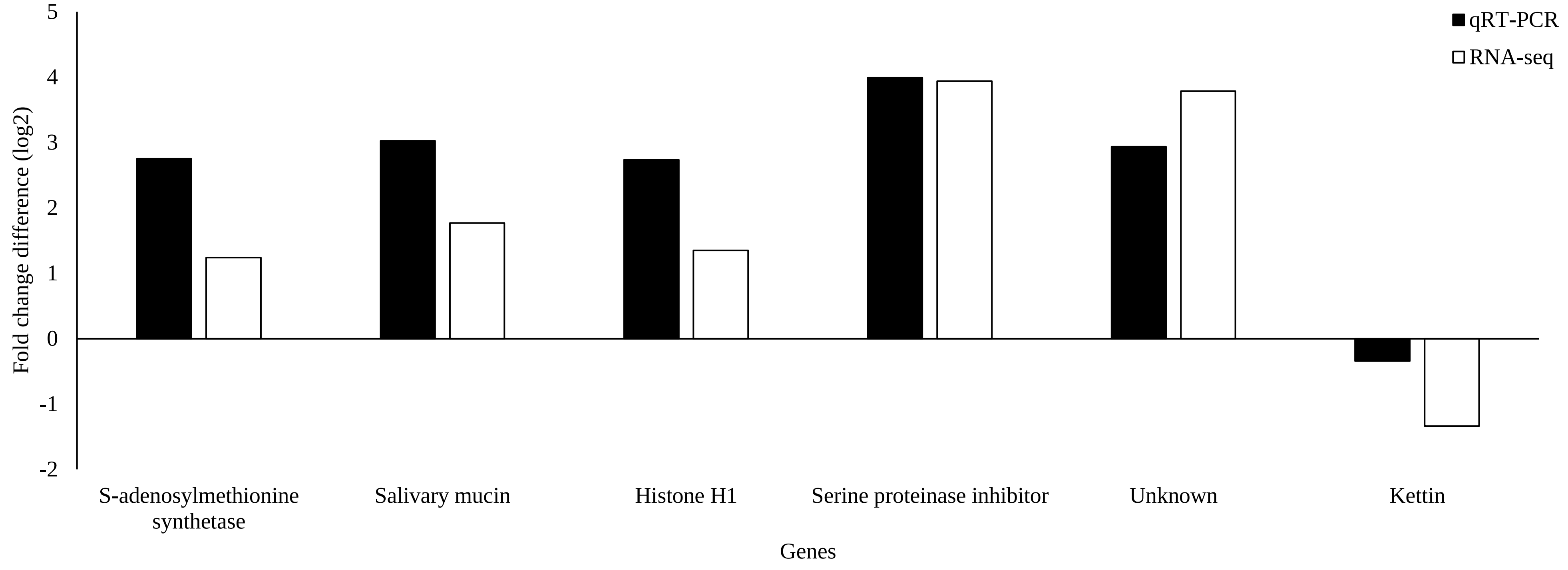

(b) *M. anisopliae* JEF-290

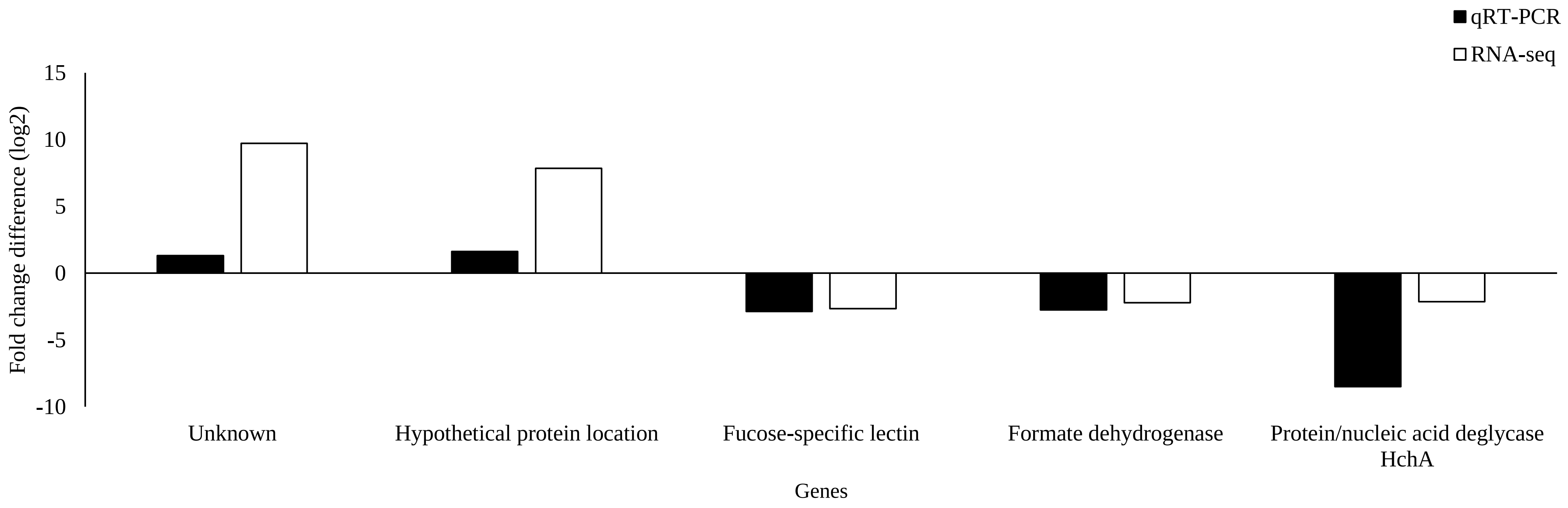

Figure S2. Validation of RNA-sequencing data using qRT-PCR.
